# Lactulose may reduce *Clostridium difficile*‐related diarrhea among patients receiving antibiotics

**DOI:** 10.1002/jgh3.12390

**Published:** 2020-07-09

**Authors:** Charles Maltz, Paul F Miskovitz, Kaveh Hajifathalian

**Affiliations:** ^1^ Division of Gastroenterology and Hepatology Weill Cornell Medical College New York New York USA

**Keywords:** *Clostridium difficile*, diarrhea, lactulose

## Abstract

**Background and Aim:**

Prebiotics are nondigestible oligosaccharides that are metabolized by colonic bacteria, resulting in a change in the pH of the colonic milieu as well as modifying the microbiome of the colon. The purpose of this retrospective study was to determine whether concomitant lactulose administration affected the *Clostridium difficile* infection rate among hospitalized adult patients receiving antibiotics.

**Methods:**

We retrospectively reviewed inpatient medical records of patients in a large teaching hospital admitted during a one‐year period. Individuals treated with antibiotic therapy during the course of their hospitalization were considered for inclusion in the study. Patients were evaluated for development of *C. difficile* infection, as well as concomitant lactulose therapy for hepatic encephalopathy. The incidence of *C. difficile* infection among patients who received lactulose and antibiotic therapy was compared with that among those who received antibiotic therapy alone.

**Results:**

Patients who received lactulose and antibiotic therapy were slightly older (*n* = 87, mean age 67) than patients who received antibiotic therapy alone (*n* = 103, mean age 60). Similar numbers of patients were males in both groups (male/female: 50/53 and male/female: 46/41). Two (2.3%) patients who received lactulose and antibiotic therapy developed *C. difficile* infection during the course of hospitalization, compared with 10 (9.7%) patients who received antibiotic therapy alone (*P* = 0.04, Fisher exact test).

**Conclusion:**

Administration of lactulose may reduce the incidence of *C. difficile*‐related diarrhea among hospitalized adult patients receiving antibiotics.

## Graphical abstract

This was an observational study that suggested that lactulose given along with antibiotics decreased the incidence of *Clostridium difficile* infection.

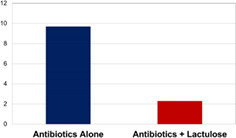

## Introduction

*Clostridium difficile* is an increasingly frequent community‐acquired infection and a significant cause of diarrhea among hospitalized and institutionalized patients. Risk factors for infection include current or recent hospitalization, proton pump inhibitor usage, advanced age, severe illness, and prior *C. difficile* infection, although antibiotic therapy is the most often implicated causative factor.^1^ In the normal situation, the gut microbiome promotes an environment that is inhospitable for *C. difficile*. However, any of the abovementioned factors can modify the normal flora and remove the barrier to *C. difficile* proliferation. For this reason, patients with *C. difficile* infection may be successfully treated with fecal transplantation. The modification of the colonic milieu by probiotics in order to prevent the growth of *C. difficile* have given inconsistent results, with the most favorable data occurring for the yeast *Saccharomyces boulardii*.^2^, ^3^

Prebiotics are nondigestible carbohydrate polymers that can be metabolized by some bacterial strains comprising the colonic flora.^4^, ^5^ Fructooligosaccharides are polymers of the ketonic carbon sugar fructose and are not digested and serve as energy sources for the bacteria in the colon. Lactulose is a synthetic disaccharide composed of fructose and galactose and has been used to treat portosystemic encephalopathy in patients with chronic liver disease and to treat patients with chronic constipation. Because there is no disaccharidase in the gastrointestinal tract to hydrolyze lactulose, it passes into the colon unchanged.^6^ There it is fermented by bacteria in the colon. The mechanisms of action in treatment of portosystemic encephalopathy have been postulated to include some combination of modification of the bacterial flora of the colon so that the growth of bifidobacteria and lactobacilli are promoted,^7^ a cathartic effect resulting in elimination of colon contents, and an acidification of luminal contents with protonation of ammonia to ammonium.^6^, ^7^, ^8^ These effects may have a deleterious effect on *C. difficile*, which is susceptible to changes in acidity, as well as to the presence of other colonic flora, including *Lactobacillus*.^9^ Two prospective studies by the same group demonstrated that although the administration of oligofructose along with standard antibiotic treatment for *C. difficile* reduced the recurrence rate of *C. difficile* diarrhea, the administration of oligofructose at the time of initial antibiotic diarrhea did not prevent *C. difficile* diarrhea.^10^, ^11^ A recent study by Agarwalla and colleagues of decompensated cirrhotics compared those with *C. difficile* infection with controls. Lactulose was associated with a decreased incidence of *C. difficile* infection.^12^

## Methods

This study was performed as a retrospective review of inpatient admissions to the medicine and surgery floors in a large academic center over the period of 1 year.

Medical records of consecutive hospital admissions during a 1 year period were reviewed to find comparable number of patients with cirrhosis who received antibiotics and lactulose and were tested for *C. difficile* during their admission, as well as a comparison group of general population of patients who received antibiotics but not lactulose, and were also tested for *C. difficile* infection during their admission.

Consecutive patients with liver cirrhosis who were admitted during the study period, received oral or intravenous antibiotics as well as lactulose (for management of hepatic encephalopathy) during their admission, and were tested for *C. difficile* infection were included in the analysis. Cirrhotic patients with previous history of *C. difficile* infection before the index admission, and those who received metronidazole, rifaximin, and/or oral vancomycin without first having been diagnosed with *C. difficile* during the index admission were excluded. A comparison cohort of general population of patients without cirrhosis who were admitted to the medicine and surgery floors, received oral or intravenous antibiotics (but not lactulose) and were tested for presence of *C. difficile* during their admission were also included in the analysis. Similar to the cirrhotic cohort, patients with prior history of *C. difficile* infection before the index admission, and those who received metronidazole, rifaximin, and/or oral vancomycin without first having been diagnosed with *C. difficile* during the index admission were excluded.

The main exposure was defined as inpatient treatment with oral lactulose. Lactulose was prescribed to manage hepatic encephalopathy in patients with cirrhosis. It was started at a dose of 20 g orally, three times daily, and was titrated as needed to achieve three loose bowel movement per 24 h. None of the patients in the lactulose group received this medication for any indication other than management of hepatic encephalopty.

The main outcome of the study was inpatient diagnosis of *C. difficile* infection based on nucleic acid amplification test using polymerase chain reaction (PCR) to detect toxigenic *C. difficile* strains in patients' stool sample, in accordance with Infectious Diseases Society of America (IDSA) guidelines. To qualify for the *C. difficile* stool test, patients had to have three episodes of diarrhea (watery stool) over a period of 24 h before the test was performed. The incidence of *C. difficile* was compared between patients who received lactulose (cirrhotic cohort), and those who did not using chi‐squared test. All tests are two‐sided with a significance level of 0.05.

## Results

**Figure 1:**
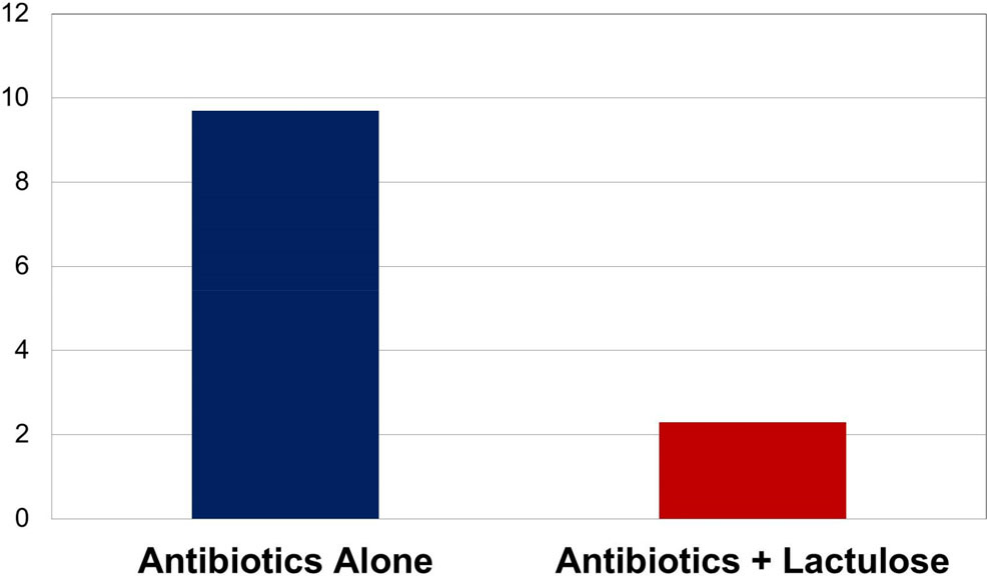
Percentage of patients who developed *Clostridium difficile* diarrhea in each group.

A total of 103 patients (50 men and 53 women) were included in the antibiotics alone cohort and 87 patients (46 men and 41 women) were included in the antibiotics with lactulose cohort (Table 1). Analysis of *C. difficile* infection rates revealed that 2 of 87 patients (2.3%) receiving both lactulose and antibiotics developed *C. difficile* infection compared with 10 of 103 patient (9.7%) receiving antibiotics alone (*P* = 0.04) (Fig. 1).

**Table 1:** Patient characteristics

	Antibiotics alone	Antibiotics + lactulose	*P*‐values
Male:Female	50:53	46:41	0.56
Average age	60	67	0.02
Cases of *Clostridium difficile*	10 (9.7%)	2 (2.3%)	0.04

## Discussion

The possible utility of prebiotics in treating or preventing *C. difficile* infection is an idea that is at more than a decade old. Xylitol is a five‐carbon sugar alcohol that is incompletely absorbed. In 1996, investigators postulating that mucosal association is an important factor influencing intestinal colonization and subsequent infection with *C. difficile* investigated the effect of xylitol on the adhesion of the *C. difficile* bacterium to colonic mucosa and discovered a dose dependent inhibition by xylitol on *C. difficile* mucosal adherence.^13^ In the same year, Japanese investigators demonstrated that decreased fecal levels of iC4‐nC6 fatty acids after lactulose supplementation may be related to suppression of growth of iC4‐nC6 fatty acid‐producing fecal organisms, especially *C. difficile*.^9^ A dose of 10 g per day has been shown in a randomized double‐blind study to increase fecal bifidobacterial counts.^5^ The dose used for hepatic encephalopathy is at least 30 g per day.

Our preliminary retrospective chart review findings suggest that concomitant lactulose therapy may help prevent the development of *C. difficile* infection in hospitalized patients receiving antibiotics. The mechanism of action of lactulose is speculative and might involve the effects on bacterial colonization (including mucosal adherence and/or the suppression of growth of iC4‐nC6 fatty acids‐producing fecal organisms), toxin production, and/or toxin activity.^5^ Although the patients in the two cohorts were similar in age, they were not identical in that the patients in the lactulose all had chronic liver disease. Our present study was not controlled for the other multiple known risk factors for *C. difficile* infection such as proton pump inhibitor usage.^14^ Finally, the group with lactulose and antibiotics may have been more likely tested for *C. difficile* than the group on antibiotics alone which may have diluted the fraction of patients on lactulose and antibiotics with *C. difficile*. However, this more frequent testing would also be expected to diagnose more cases of *C. difficile* in patients who otherwise would not have been tested. Our findings, along with the others cited, may spur future research in the form of a study with attention to dosing and duration of concomitant therapy with the prebiotic lactulose and the composition of the fecal microbiome in those patients who received lactulose and who did and did not develop *C. difficile* diarrhea.
